# Comparison of the Standard vs. Thoracoscopic Extrapleural Modification of the Nuss Procedure—Two Centers’ Experiences

**DOI:** 10.3390/children9040557

**Published:** 2022-04-14

**Authors:** Miloš Pajić, Damjan Vidovič, Radoica Jokić, Jelena Antić, Nenad Čubrić, Ivana Fratrić, Svetlana Bukarica, Aleksandar Komarčević, Marina Milenković

**Affiliations:** 1 Clinic for Pediatric Surgery, Institute for Child and Youth Healthcare of Vojvodina, Hajduk Veljkova 10, 21000 Novi Sad, Serbia; milos.pajic@mf.uns.ac.rs (M.P.); radoica.jokic@mf.uns.ac.rs (R.J.); jelena.antic@mf.uns.ac.rs (J.A.); svetlana.bukarica@mf.uns.ac.rs (S.B.); aleksandar.komarcevic@mf.uns.ac.rs (A.K.); marina.milenkovic@mf.uns.ac.rs (M.M.); 2 Faculty of Medicine, University of Novi Sad, Hajduk Veljkova 3, 21000 Novi Sad, Serbia; 3 Department of Thoracic Surgery, University Medical Centre Maribor, Ljubljanska ulica 5, 2000 Maribor, Slovenia; damjan.vidovic@ukc-mb.si (D.V.); nenad.cubric@gmail.com (N.Č.)

**Keywords:** pectus excavatum, Nuss procedure, thoracoscopic extrapleural modification, children

## Abstract

Pectus excavatum is the most common congenital anterior chest wall deformity, with an incidence of 1:400 to 1:1000. Surgical strategy has evolved with the revolutionary idea of Donald Nuss, who was a pioneer in the operative correction of this deformity using minimally invasive surgery. The aim of this paper is to compare the preliminary results of pectus excavatum repair in two University Centers with a moderate number of patients using the standard Nuss procedure and its modification, the extrapleural thoracoscopic approach. The statistical analysis showed no significant difference for the patient’s age (14.52 ± 3.70 vs. 14.57 ± 1.86; *p* = 0.95) and the CT Haller index (4.17 ± 1.58 vs. 3.78 ± 0.95; *p* = 0.32). A statistically significant difference was noted for the duration of a pectus bar implant (2.16 ± 0.24 vs. 2.48 ± 0.68; *p* = 0.03) between the Maribor and Novi Sad Center. We report 14 complications (28%), including dislocation of the pectus bar (10%), pleural effusion (8%), wound inflammation (6%), pericarditis (2%) and an allergic reaction to the pectus bar (2%). Standard and thoracoscopic extrapleural Nuss procedures are both safe and effective procedures used to correct a pectus excavatum deformity. The choice of surgical procedure should be made according to a surgeon’s reliability in performing a particular procedure. Our study found no advantages of one procedure over the other.

## 1. Introduction

Pectus excavatum is the most common congenital anterior chest wall deformity, with an incidence of 1:400 to 1:1000 [1]. Apart from this deformity, pectus carinatum, Poland syndrome and sternal defects are usually listed as anterior chest wall deformities. The first reports of a pectus excavatum deformity date back to the XVI century [2]. Nowadays, one of the most-used diagnostic procedures for a pectus excavatum deformity is computed tomography (CT) to determine the Haller index (the ratio between the latero–lateral and antero–posterior diameter). Numerous attempts have been made to decrease the level of radiation by using CT with minimal radiation (single-slice CT, etc.). A Haller index value of more than 3.25 and more than 3.5 usually implies a moderate and severe chest wall deformity, respectively. Surgery is indicated in the majority of these patients [3]. The most popular technique used in the previous period was invented by Mark Ravitch in 1948. The operation consists of complete resection of all sternal attachments, a significant loss of blood, impressive correction of the deformity and respective results [4]. Surgical strategy has evolved with the revolutionary idea of Donald Nuss (1987), who was a pioneer in operative correction of this deformity using minimally invasive surgery (Minimal Invasive Repair of the Pectus Excavatum: MIRPE). This technique is without a large incision (bilateral 3–4 cm incision), cartilage resection and sternal osteotomy [4]. Soon, it became the first-line treatment option in many pediatric surgical centers because of its efficacy, safety, shorter hospitalization period and faster return to everyday activities. However, some complications after the Nuss procedure have been mentioned, such as wound infections, pneumothorax, pleural effusions, hematothorax, bar displacement, myocarditis or Dressler syndrome [5,6], etc. Serious complications such as cardiac perforation have also been published [7]. During the last period, numerous modifications of the Nuss procedure have been described. Modifications were didactically divided into groups depending on the selection of patients; patient positioning; the place of operative incision; the shape, size and number of bars used; fixation; bar passage; modifications of the bars’ stabilization, etc. Among the above-mentioned modifications, one that exceeds others in its expectations is the extrapleural approach. This operation includes bilateral thoracic skin incisions and blunt finger dissection with the creation of an extrapleural tunnel for placement of the pectus bar. This technique is also academically divided into a non-thoracoscopic and thoracoscopic extrapleural modification. 

The aim of this paper is to compare the preliminary results of pectus excavatum repair in two University Centers with a moderate number of patients using the standard Nuss procedure and its modification, the extrapleural thoracoscopic approach. 

## 2. Material and Methods

### 2.1. Patients

Medical data from 50 patients (21 from the Institute for Child and Youth Health Care of Vojvodina, Serbia, and 29 patients from the University Medical Center in Maribor, Slovenia) who underwent the minimally invasive surgical procedure for correction of a pectus excavatum deformity between 2006 and 2017 were collected and analyzed. Those were the first patients operated on in both centers, so this data could serve as a “learning curve” process. 

Institutional Review Board: This retrospective study was approved by the Ethical Committee of the University Medical Center in Maribor, Slovenia, and the Institute for Child and Youth Health Care of Vojvodina, Serbia (code 133-4 from 13 January 2022).

Inclusion criteria: This study first included 21 patients of both genders with a pectus excavatum deformity that underwent the minimally invasive Nuss procedure at the Institute for Child and Youth Health Care of Vojvodina, Serbia, and 29 patients with a pectus excavatum deformity who underwent the extrapleural thoracoscopic modification of this procedure at the University Medical Center in Maribor, Slovenia. All patients and their parents or guardians signed informed consent. They were checked by a pediatric pulmonologist and cardiologist before the surgery, and additional tests were performed if needed. 

The indication for operation was a pectus excavatum deformity with a CT Haller index of more than 3.25, children with severe clinical manifestation of this deformity with cardiopulmonary symptoms in whom this index was even less than 3.25, a clinically progressive deformity and/or intolerance to exercise. 

Exclusion criteria: patients with a severe form of a pectus excavatum deformity and/or an asymmetrical deformity who were referred for another surgical approach (for example, the Ravitch procedure); patients with a combination of a pectus excavatum and pectus carinatum chest wall deformity and patients with an incomplete dataset.

### 2.2. The Expected Outcome of the Study

The primary expected outcome of this study was to compare the age, CT Haller index and duration of the pectus bar implant in patients operated on using the standard Nuss procedure in one medical center and its modification, the extrapleural thoracoscopic approach, in the other medical center. 

The secondary expected outcome of the study was to compare the number and structure of complications noted in the center using the standard Nuss procedure and the extrapleural thoracoscopic approach.

### 2.3. Study Protocol

This study was designed as a retrospective observational study. Medical data, such as the age and gender of the patients, the CT Haller index preoperatively, date of the surgery for correction of the pectus excavatum deformity, date of the surgery for extraction of the pectus bar and complications for each patient were recorded.

### 2.4. Description of Surgery

**Figure 1:**
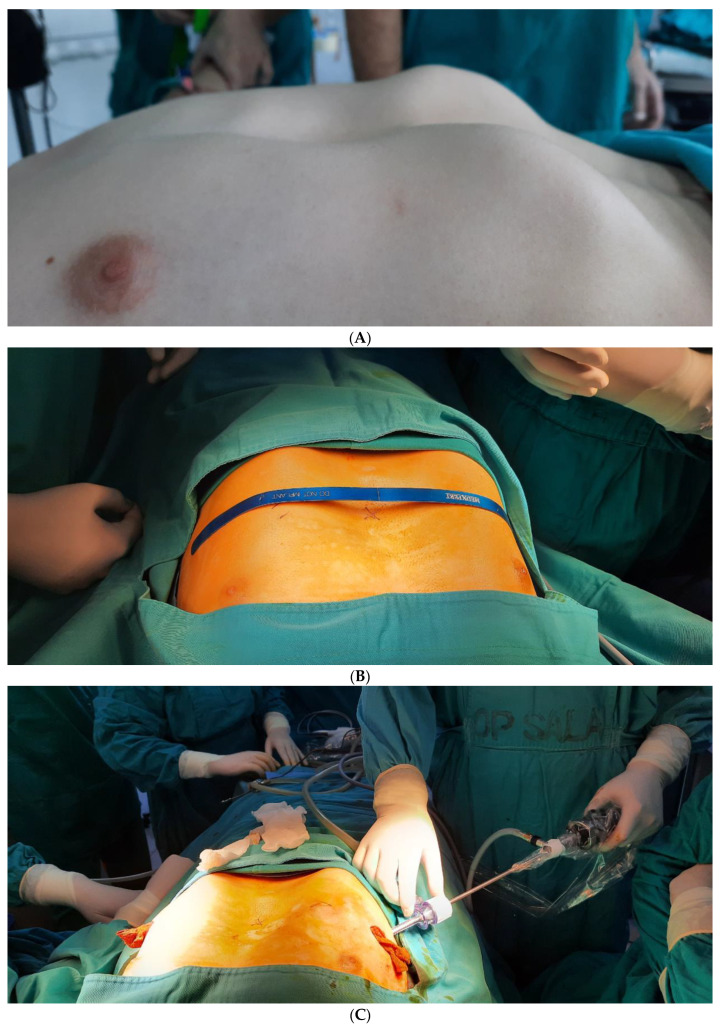

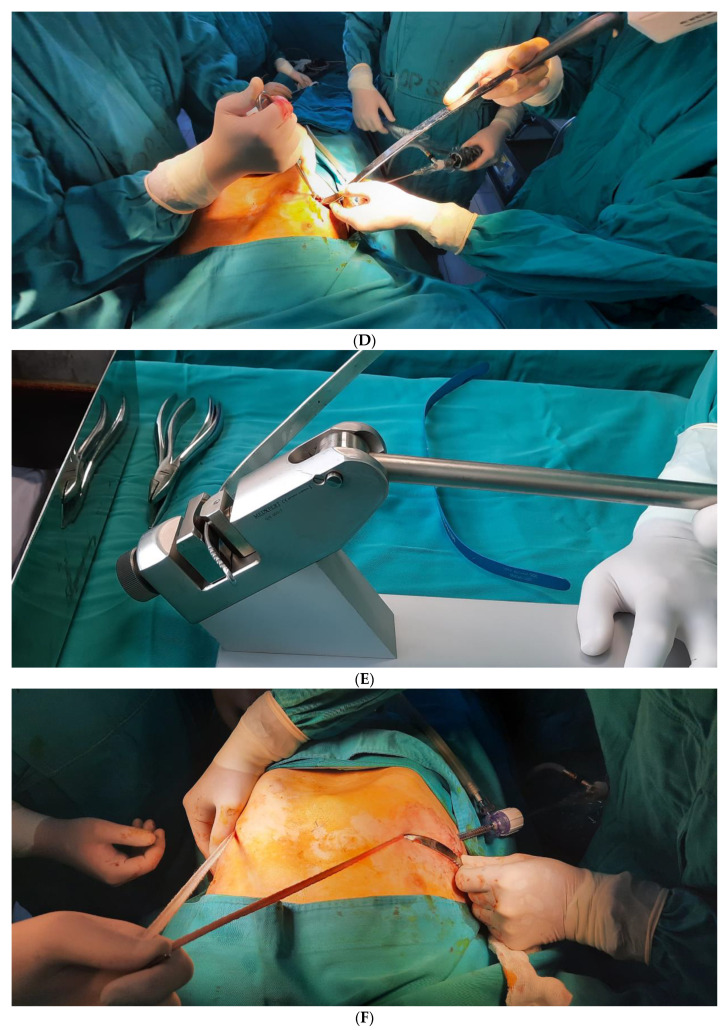
(**A**) Pectus excavatum deformity before the surgery. (**B**) Configurated and modified plastic model of the pectus bar. (**C**) The use of a thoracoscopic camera during the Nuss procedure. (**D**) The use of the Lorenzo introducer. (**E**) Shaping of the metal/titanium pectus bar based on the plastic model. (**F**) The bar was flipped for its final placement with its convexity to the sternum.

The operative technique for patients in Novi Sad was the standard Nuss procedure with thoracoscopic visualization and control during passing of the pectus bar. The patient was in a supine position with their arms abducted (Figure 1A). Then, the distance from one midaxillary line to the other was measured, and 2.5 cm was subtracted for the exact length of the pectus bar. The plastic model of the pectus bar was then configured and modified in order to correct the deformity of the thorax (Figure 1B). Then, two lateral planned incisions, 3–4-cm-long, were marked on the imagined horizontal line which connects the deepest part of the sternum, the thorax ridges of both sides and the planned incisions. The space for the pectus bar and stabilizer was created under the pectoral muscles. A thoracoscopic camera was inserted two intercostal spaces below the planned pass of the bar to allow visibility and, specially, to check passing of the bar below the sternum and above the pericardium (Figure 1C). An artificial pneumothorax was achieved by insufflation of CO_2_ (2–7 mmHg, which depends on the age of the patient, their weight, the shape of the thorax and the characteristics of the anomaly). Under thoracoscopic control, a Lorenzo introducer (a specially created titanium guide) was inserted into the thoracic cavity below the deepest part of the sternal deformity at the level of the future placement of the pectus bar (Figure 1D). When the introducer was pushed out on the contralateral side, it was possible to predict the correction of the deformity. Two umbilical tapes (cotton straps) were attached to the introducer and pulled back. The first one was used as security, and the second cotton strap was attached to the previously shaped (Figure 1E) metal/titanium pectus bar, based on the plastic model (Pectus Support Bar, Biomet Microfixation, Inc., Jacksonville, FL, USA, SAD). It was slowly pulled back on the contralateral side, with its concavity below the sternal deformity. Particularly designed flippers (rotators) were used to rotate the bar with its convexity to the sternum, providing immediate correction of the pectus excavatum deformity (Figure 1F). The bar was then fixated by inserting it into the stabilizer (Pectus elongated Stabilizer, Biomet Microfixation, Inc., Jacksonville, FL, USA, SAD) and secured by tying surgical steel wire around the ribs. We used the stabilizer only on one side and fixation of the pectus bar on the contralateral side using two double polyester polyfilament non-resorptive sutures. Here, this fixation was performed under the pectoral muscle to the nearest ribs as well. Thoracic drains were not used if complete evacuation of the insufflated CO_2_ was maintained and lung re-expansion was tracked with a thoracoscope until the end of the surgery. 

The operative technique used in the University Medical Center in Maribor was the thoracoscopic extrapleural modification of the Nuss procedure. The procedure began with the insertion of a 5 mm trocar into the right pleural cavity and with insufflation of CO_2_ (5–6 mmHg) for an artificial pneumothorax. Under thoracoscopic guidance, the extrapleural tunnel was created using a blunt dissection from both sides. The special surgical instrument (dissector) was guided along the path between the pleura and the posterior side of the sternum until it reached the contralateral side. After the tunnel was made, two umbilical tapes (cotton straps) were attached to the dissector and pulled back. The first one was used as security, and the second umbilical tape (cotton strap) was attached to the previously shaped metal/titanium pectus bar and slowly pulled back on the contralateral side, with its concavity below the sternal deformity. Specially designed flippers (rotators) were used to rotate the bar with its convexity to the sternum, providing correction of the pectus excavatum deformity. Stabilizers were attached to the one end of the bar and secured to the ribs with nonabsorbable sutures. Practically, this modification differed from the standard Nuss procedure in the dissection and positioning of the pectus bar above the pleura. 

### 2.5. Follow-Up

After surgery, the child was placed in the intensive care unit for around 3 days. During this period, the child was given intravenous pain medication according to his/her level of pain, and respiratory physical treatment was started. In the last period, we started using thoracic epidural anesthesia for pain management. At the start of the procedure, clinical hospitalization lasted for about 7 days, and usually 3 to 4 weeks were necessary for returning to everyday activities. There is a tendency to decrease the length of hospitalization. Outpatient visits were scheduled after the patient was discharged home, and late complications were noted in each of these controls. The extraction of the pectus bar was usually scheduled after 3 ± 1 years. 

### 2.6. Statistical Analysis

Medical data were analyzed using the SPSS Statistics 20.0 (IBM Corporation, Armonk, NY, USA) and Microsoft Excel 2010 program (Microsoft Corporation, Redmond, WA, USA). Descriptive parameters and a parametric (*t*-test) and nonparametric (Mann–Whitney U test) tests were used to determine the difference between the groups and its significance.

## 3. Results

Medical data of 50 patients, 21 in Novi Sad and 29 in Maribor, were collected and analyzed in this study. 

According to the statistical analyses of the patient’s age (*p* = 0.95) and CT Haller index (*p* = 0.32), we did not find a statistically significant difference between these two groups (Maribor and Novi Sad). This difference was noted when comparing the duration of the pectus bar implant (*p* = 0.03) in Maribor and Novi Sad (Table 1).

As seen in Table 2, the mean age for male patients in both centers was around 15 years (slightly more in Maribor than in Novi Sad). Unlike in male patients, female patients were younger at the time of operation in Maribor than in Novi Sad (11.80 years vs. 14.50 years). The age difference among these two centers in relation with gender was not statistically significant (*p* = 0.55 and *p* = 0.35 in male and female patients, respectively).

**Figure 2:**
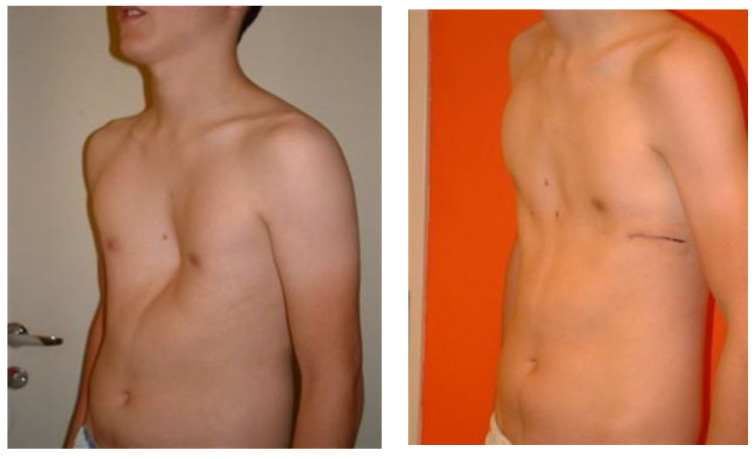
A boy with a pectus excavatum deformity before the surgery and after the standard Nuss procedure.

**Figure 3:**
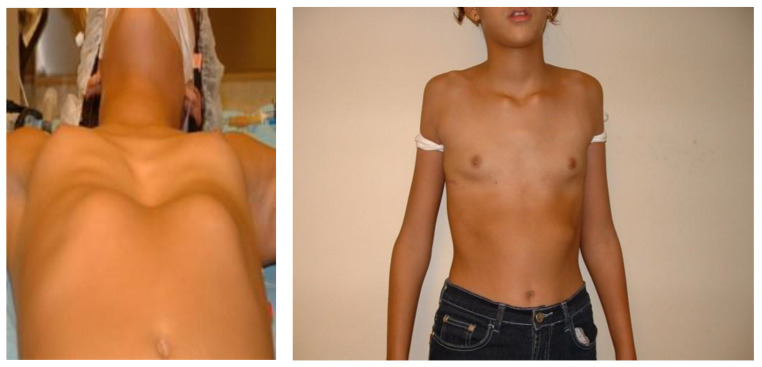
A girl with a pectus excavatum deformity before the surgery and after the standard Nuss procedure.

Higher values of the CT Haller index were noted in Novi Sad in comparison to Maribor when we analyzed only female patients, but this difference was not statistically significant (*p* = 0.23). On the contrary, higher values of the CT Haller index in male patients were noted in Maribor, with no statistical significance (*p* = 0.11). The appearance of male and female patients before and after the surgery is shown in Figure 2 and Figure 3. 

The duration of the pectus bar implant was more than 2 years in Maribor and in Novi Sad, but a slightly longer period between the implantation and extraction of the pectus bar was noted in Novi Sad in both genders.

All patients were followed until pectus bar removal, and complications were noted during this time interval.

All procedures were successful with several postoperative complications (14 in both centers and 28% in total), such as dislocation of the pectus bar (10%), pleural effusion (8%), wound inflammation (6%), pericarditis (2%) and an allergic reaction to the pectus bar (2%). The distribution of these complications in two analyzed centers is shown in Table 3 without a statistically significant difference in the outcome and complication rate (*p* = 0.94).

### Management of Complications

Most of the complications in our study were mild, with the need for readmission of three patients in Novi Sad (wound abscess, dislocation of the pectus bar and pleural effusion with pericarditis). 

The patient with a wound abscess (pseudomonas aeruginosa) was treated with drainage and a parenteral antibiotic (gentamicin) for 6 days and continued with peroral use of ciprofloxacin for the next 5 days.

One patient with dislocation of the pectus bar needed reoperation within 6 weeks from the first operation. The postoperative course was uneventful.

The patient with pericarditis and pleural effusion reacted well on a conservative treatment (antibiotic and corticosteroid therapy) without the need for thoracal drainage.

Patients with wound inflammation in Maribor were treated with regular dressings and antibiotics. In patients with a significant pleural effusion, thoracic drainage was used (Trocar catheter, Dahlhausen, Koeln, Germany) for the two to three days.

One patient with dislocation of the pectus bar in Maribor needed reoperation for repositioning of the bar.

A 30-day readmission rate from discharge was used in our study as a quality-of-care indicator. The total number of readmissions in the 30-day postoperative period in Novi Sad in the two years in which we had complications related to the standard Nuss procedure was 122 out of 3313 (3.38%) and 115 out of 3086 (3.73%). Complications after the standard Nuss procedure at Novi Sad were the cause for readmission in 0.87% and 0.82% of patients, while in Maribor, none of the patients were rehospitalized in the 30-day postoperative period after the extrapleural modification of Nuss procedure.

## 4. Discussion

After the first presentation of the Nuss procedure with impressive results, this minimally invasive technique was soon accepted as the standard surgery for pectus excavatum repair. The initial, original Nuss procedure did not use thoracoscopy, but quickly, this kind of visual control was introduced. Unilateral thoracoscopy is slightly more dominant than bilateral thoracoscopy and is considered a standard Nuss procedure [8,9,10].

Our experience with the standard Nuss procedure dates back to 2006, when we started this procedure in collaboration with the experts from Graz, Trieste and Tel Aviv. Their help and support overall, especially with patients in whom the deformity was severe and we had to place two pectus bars in order to correct the deformity, was significant. For this study, we investigated the treatment of pectus excavatum in the first 21 patients in Novi Sad using the standard Nuss procedure and in 29 patients in Maribor using the extrapleural modification of this procedure.

In 2002, the extrapleural approach was introduced in the practice as reported by Schaarschmidt [11]. The proposed and reported benefits of this technique included the prevention of pericardium injury, the prevention of pleural adhesions and lung injury by not making direct contact with the lung and even the prevention of compression of the internal mammary arteries. It should be emphasized here that this technique cannot prevent cardiac injury as the pleura is not strong enough against the precise amount of force needed to pass the dissector [10]. Furthermore, it is arguable if compression of the mammary arteries increases the risk of hemorrhagic complications in pediatric patients at all [12].

Apart from modifications in the operative techniques, there are several modifications in diagnostic procedures as well. The most important consists of decreasing the level of radiation through the introduction of a single slice CT with minimal radiation for the patient or the use of noninvasive methods, such as MRI. While the conventional method of determining the Haller index is in the inspiration phase, our previous study [2] suggests that an end-expiration measurement is more accurate. An example of this method is seen in patients with a CT Haller index below the surgical indications in the inspiration phase (e.g., 2.45). Further studies are needed to support this assumption.

On the other hand, between the new surgical procedure, computer-aided design (CAD) is especially interesting. It consists of 3D CT planning and construction of a custom-made implant which is used to fill in the anterior chest wall depression [13]. It is a less invasive procedure, but it consists of a relatively large incision in the middle of the chest, and it does not resolve the pressure on the cardiovascular system and lungs. Further modifications with periareolar incisions have now been described [14].

Although many children with pectus excavatum would have little to no cardiorespiratory symptoms, they would benefit through long-term cardiopulmonary function improvement [15]. A cardiologic and pulmonologist consultation (pre- and post-operative) is considered a standard protocol at our Institutions.

Both centers involved in this study were operating on patients with a median age of 15 years, which is in accordance with the literature findings [16]. Practically, most centers do not operate on patients younger than 10 years of age because of the high recurrence rate [17].

Our study showed a mean Haller index of 4.17 in Maribor and of 3.78 in Novi Sad, with increased levels of this value in male patients in Maribor (4.32). However, a higher level of the CT Haller index was reported in female patients in Novi Sad (4.31). The higher level of the Haller index in female patients could be explained by their better cardiopulmonary function and exercise tolerance despite a worse deformity [18]. This suggests that female patients seek medical attention later than male patients or only when their deformity is really severe. Secondly, this deformity is challenging to recognize in female patients due to breast development and changes in puberty.

The period between the implantation and extraction of the pectus bar in our study was 2.16 years in Maribor and 2.48 years in Novi Sad, which is in accordance with the literature findings [19,20]. Most authors suggest removal of the pectus bar 2–4 years after its implantation, as earlier removal is associated with a higher recurrence rate. Late removal of the pectus bar could be difficult to perform due to a significant ossification [19]. Despite not having a unique guideline on the exact timing of pectus bar extraction, most authors agree to remove the bar after the fracture healing process is usually completed. This happens faster in children than in adults, in whom this process lasts for 4.3 years on average [21].

Although all procedures were successful, we reported several postoperative complications (14 in total), which accounted for 28% of patients. Our study showed a similar degree of complications as reported in the literature [22,23,24], but in our study, the complications were usually mild.

Both centers analyzed in our study noted dislocation of the pectus bar (10.34% in Maribor and 9.52% in Novi Sad). Mostly, the patients with dislocation did not need reoperation, but in one patient, reoperation with repositioning of the pectus bar was performed in each center. The literature suggests that dislocation of the pectus bar happens in up to 33% of cases [25,26], but dislocation with reoperation has been noted in only up to 5% of cases [27,28]. In total, we reported 10% of patients with dislocation of the pectus bar, with only 4% needing operative revision.

Mild wound inflammation was noted in both centers (6.90% in Maribor, 4.76% in Novi Sad and 6% in total), which resolved with regular dressing and antibiotics without the need for extraction of the pectus bar.

Furthermore, a common complication was pleural effusion (10.34% in Maribor and 4.76% in Novi Sad). The total of 8% of patients with pleural effusions reported in our study is in accordance with the literature findings (up to 10%) [26,29,30]. Unlike as expected, we noted less pleural effusions after the standard Nuss procedure compared to the extrapleural thoracoscopic modification. In patients with a significant pleural effusion, thoracic drainage was used (Trocar catheter, Dahlhausen, Koeln, Germany) for the two to three days. Pleural effusion in the standard Nuss procedure happens probably due to an unspecific inflammatory reaction of the parietal pleura with increased permeability of the pleural membrane. Additionally, injuries of small vessels on the parietal pleura caused by blind dissection in the standard Nuss procedure could have led to pleural effusion. Our study showed more pleural effusions in the extrapleural modification of the Nuss procedure, probably due to irritation of the pleura from the pectus bar placement. Transient pleural effusions in both procedures could be also explained by the empty space created by the elevation of the sternum and atelectasis [31].

We reported one case of pericarditis (0% in Maribor, 4.76% in Novi Sad and 2% in total) that was presented and published [6] and one allergic reaction to the pectus bar (0% in Maribor, 4.76% in Novi Sad and 2% in total). The patient with the allergic reaction to the pectus bar reacted well on symptomatic treatment, and there was no need for extraction of the pectus bar.

If we divide our complete experience into two periods, we unexpectedly experienced more complications in the last 6 years, probably due to acceptance and the inclusion of more complex cases for the Nuss procedure.

A 30-day readmission rate was used as an indicator for the quality-of-care in our study. Complications after the standard Nuss procedure account for 0.8% of readmissions and are considered as an indicator of good clinical practice according to the literature [32].

Our study showed no statistically significant difference (*p* = 0.94) in the outcomes and complication rates between these two procedures (standard and thoracoscopic extrapleural modification of the Nuss procedure), which implies that both procedures can be used as they are safe and effective. Despite all the innovations and modifications of Nuss procedure, we are still facing similar levels of complications worldwide [22,23,24,25,26,27,28,29,30].

The limitations of our study include its retrospective design and small number of patients. A prospective study with the aim of comparing two homogenous groups of a larger number of patients is needed to further evaluate the efficiency and safety of the standard versus the thoracoscopic extrapleural modification of the Nuss procedure.

## 5. Conclusions

The standard and the thoracoscopic extrapleural modification of the Nuss procedure are both safe and effective procedures used to correct a pectus excavatum deformity. Our study found no advantages of one procedure over the other.

The similar range of complications with good results suggest that the choice of the surgical procedure should be made according to the surgeon’s comfort with the procedure, which leads to less complication rates and better final results. The choice of the surgical procedure should be made according to the surgeon’s reliability in a particular procedure based on the adopted and practiced surgical method.

## Figures and Tables

**Table 1:** Frequency values for age, the CT Haller index and duration of the pectus bar implant in Maribor (Mb) and Novi Sad (NS).

	Maribor			Novi Sad			*p* Value
	Mean	Std. Deviation (SD)	Median	Mean	Std. Deviation (SD)	Median	
Age (years)	14.52	3.70	15.00	14.57	1.86	15.00	0.95
Haller index	4.17	1.58	3.77	3.78	0.95	3.76	0.32
Duration of the pectus bar implant (years)	2.16	0.24	2.10	2.48	0.68	2.00	0.03

**Table 2:** Frequency values for age, the CT Haller index and duration of the pectus bar implant for each gender in Maribor and Novi Sad.

		Maribor				Novi Sad				*p* Value
		Mean	SD	Min	Max	Mean	SD	Min	Max	
Age (years)	Male	15.08	2.75	8.00	21.00	14.60	1.92	10.00	18.00	0.55
	Female	11.80	6.42	5.00	21.00	14.50	1.87	12.00	17.00	0.35
Haller index	Male	4.32	1.69	2.45	9.70	3.57	0.63	2.50	5.02	0.11
	Female	3.46	0.50	2.90	4.14	4.31	1.43	2.62	6.90	0.23
Duration of the pectus bar implant (years)	Male	2.18	0.26	2.00	3.00	2.40	0.63	2.00	4.00	0.14
	Female	2.08	0.13	2.00	2.30	2.67	0.82	2.00	4.00	0.15

**Table 3:** Frequency and structure of complications in Maribor and Novi Sad.

Complications	Maribor	Novi Sad	*p* Value
Dislocation of the pectus bar	3 (10.34%)	2 (9.52%)	0.92
Pleural effusion	3 (10.34%)	1 (4.76%)	0.47
Wound inflammation	2 (6.90%)	1 (4.76%)	0.75
Pericarditis	/	1 (4.76%)	
Allergic reaction to the pectus bar	/	1 (4.76%)	
Total	8 (27.59%)	6 (28.57%)	0.94

## Data Availability

All source data included in this paper are available at the Institute for Child and Youth Healthcare of Vojvodina.

## References

[1] Abid I., Ewais M.M., Marranca J., Jaroszewski D.E. (2017). Pectus Excavatum: A Review of Diagnosis and Current Treatment Options. J. Osteopat. Med..

[2] Pajic M. (2016). Clinical Significance of the Method for Thoracic Indices Assessment in Diagnosing and Treatment of Pectus Excavatum in Children. Ph.D. Thesis.

[3] Sujka J.A., Peter S.D.S. (2018). Quantification of pectus excavatum: Anatomic indices. Semin. Pediatr. Surg..

[4] Mao Y.Z., Tang S., Li S. (2017). Comparison of the Nuss versus Ravitch procedure for pectus excavatum repair: An updated meta-analysis. J. Pediatr. Surg..

[5] Schalamon J., Pokall S., Windhaber J., Hoellwarth M.E. (2006). Minimally invasive correction of pectus excavatum in adult patients. J. Thorac. Cardiovasc. Surg..

[6] Jokic R., Antic J., Marinkovic S., Bukarica S. (2012). Postpericardiotomy syndrome as a complication of Nuss procedure—Case report. Healthmed.

[7] Castellani C., Schalamon J., Saxena A.K., Höellwarth M.E. (2008). Early complications of the Nuss procedure for pectus excavatum: A prospective study. Pediatr. Surg. Int..

[8] Nuss D., Kelly R.E., Croitoru D., Katz M. (1998). A 10-year review of a minimally invasive technique for the correction of pectus excavatum. J. Pediatr. Surg..

[9] Peter S.D.S., Sharp S.W., Ostlie D.J., Snyder C.L., Holcomb G.W., Sharp R.J. (2010). Use of a subxiphoid incision for pectus bar placement in the repair of pectus excavatum. J. Pediatr. Surg..

[10] Notrica D.M. (2018). Modifications to the Nuss procedure for pectus excavatum repair: A 20-year review. Semin. Pediatr. Surg..

[11] Schaarschmidt K., Kolberg-Schwerdt A., Lempe M., Schlesinger F., Bunke K., Strauss J. (2005). Extrapleural, submuscular bars placed by bilateral thoracoscopy—A new improvement in modified Nuss funnel chest repair. J. Pediatr. Surg..

[12] Kovács T., Pásztor G., Rieth A. (2021). Internal Mammary Artery Compression After Pectus Excavatum Repair Does Not Increase Risk of Hemorrhagic Complications in Pediatric Patients. Front. Pediatr..

[13] Chavoin J.P., Grolleau J.L., Moreno B., Brunello J., Andre A., Dahan M., Garrido I., Chaput B. (2016). Correction of Pectus Excavatum by Custom-Made Silikone Implants: Contribution of Computed-Aided Design Reconstruction. A 20-Year Experience and 401 cases. Plast. Reconstr. Surg..

[14] Innocenti A., Ciancio F., Melita D., Francesco M., Portincasa A., Parisi D., Dreassi E., Innocenti M. (2017). Periareolar Access for Pectus Excavatum Correction with Silicone Implants: A New Method to Minimize Postoperative Scars—Review of the Literature, Considerations and Statistical Analysis of Clinical Outcomes. Aesthetic Plast. Surg..

[15] Chen Z., Amos E.B., Luo H., Su C., Zhong B., Zou J., Lei Y. (2012). Comparative pulmonary functional recovery after Nuss and Ravitch procedures for pectus excavatum repair: A meta-analysis. J. Cardiothorac. Surg..

[16] Kelly R.K. (2018). Modifications and Further Development of the Original Nuss Procedure: Blessing or Curse?. Eur. J. Pediatr. Surg..

[17] Kuyama H., Uemura S., Yoshida A. (2020). Recurrence of pectus excavatum in long-term follow-up after the Nuss procedure in young children based on the radiographic Haller index. J. Pediatr. Surg..

[18] Berazaluce A.M.C., Jenkins T.M., Garrison A.P., Hardie W.D., Foster K.E., Alsaied T., Tretter J., Moore R.A., Fleck R.J., Garcia V.F. (2020). The chest wall gender divide: Females have better cardiopulmonary function and exercise tolerance despite worse deformity in pectus excavatum. Pediatr. Surg. Int..

[19] Haecker F.M., Hebra A., Ferro M. (2021). Pectus bar removal—Why, when, where and how. J. Pediatr. Surg..

[20] Kelly R.E., Obermeyer R.J., Goretsky M.J., Kuhn M.A., Frantz F.W., McGuire M.M., Duke D.S., Daniel A., Nuss D. (2020). Recent Modifications of the Nuss Procedure: The pursuit of safety during the minimally invasive repair of pectus excavatum. Ann. Surg..

[21] Hsieh M.-S., Tong S.-S., Wei B.-C., Chung C.-C., Cheng Y.-L. (2020). Minimization of the complications associated with bar removal after the Nuss procedure in adults. J. Cardiothorac. Surg..

[22] Jukić M., Mustapić I., Šušnjar T., Pogorelić Z. (2021). Minimally Invasive Modified Nuss Procedure for Repair of Pectus Excavatum in Pediatric Patients: Single-Centre Retrospective Observational Study. Children.

[23] Yu S.-P., Lai P.-S., Pan C.-T., Huang P.-M. (2021). Comparison of several alternatives for the management of severe pectus excavatum in the Nuss procedure. Asian J. Surg..

[24] Pilegard H. (2015). Nuss technique in pectus excavatum: A mono-institutional experience. J. Thorac. Dis..

[25] Fan Y.-J., Lo P.-C., Hsu Y.-Y., Tzeng I.-S., Wei B.-C., Cheng Y.-L. (2021). A retrospective study on the impact of bar flipping on the recurrence of pectus excavatum after the Nuss procedure. J. Cardiothorac. Surg..

[26] Hebra A. (2018). Minor and Major Complications Related to Minimally Invasive Repair of Pectus Excavatum. Eur. J. Pediatr. Surg..

[27] Park H.J., Kim K.S., Moon Y.K., Lee S. (2015). The bridge technique for pectus bar fixation: A method to make the bar un-rotatable. J. Pediatr. Surg..

[28] Kelly R.E., Mellins R.B., Shamberger R.C., Mitchell K.K., Lawson M.L., Oldham K.T., Azizkhan R.G., Hebra A., Nuss D., Goretsky M.J. (2013). Multicenter Study of Pectus Excavatum, Final Report: Complications, Static/Exercise Pulmonary Function, and Anatomic Outcomes. J. Am. Coll. Surg..

[29] Nguyen T.-M., Le V.-T., Nguyen H.-U., Pham H.-L., Phung H.-S.D., Vu N.-T., Nguyen V.-A., Do N.-K., Vu K.-D., Vo H.-L. (2021). An Initial 5-Year Single-Center Experience of 365 Patients Undergoing the Video-Assisted Thoracoscopic Surgery for Nuss Procedure for Pectus Excavatum in Resource-Scare Setting. Front. Surg..

[30] Anbarasu C.R., Mehl S.C., Sun R.C., Portuondo J.I., Espinoza A.F., Whitlock R.S., Shah S.R., Rodriguez J.R., Nuchtern J.G., Minifee P.K. (2021). Variations in Nuss Procedure Operative Techniques and Complications: A Retrospective Review. Eur. J. Pediatr. Surg..

[31] Cheng Y.-L., Lin C.-T., Wang H.-B., Chang H. (2014). Pleural Effusion Complicating after Nuss Procedure for Pectus Excavatum. Ann. Thorac. Cardiovasc. Surg..

[32] Jukić M., Antišić J., Pogorelić Z. (2021). Incidence and causes of 30-day readmission rate from discharge as an indicator of quality of care in pediatric surgery. Acta Chir. Belg..

